# Effect of lactic acid bacteria and yeast supplementation on anti-nutritional factors and chemical composition of fermented total mixed ration containing cottonseed meal or rapeseed meal

**DOI:** 10.5713/ab.21.0270

**Published:** 2021-09-15

**Authors:** Hassan Ali Yusuf, Minyu Piao, Tao Ma, Ruiying Huo, Yan Tu

**Affiliations:** 1 Institute of Feed Research of Chinese Academy of Agricultural Sciences, Key Laboratory of Feed Biotechnology of the Ministry of Agriculture and Rural Affairs, Beijing 100081, China; 2 Faculty of Veterinary Medicine and Animal Husbandry, Somali National University, P.O Box 15 Mogadishu, Somalia

**Keywords:** Cottonseed Meal, Free Gossypol, Glucosinolates, Lactic Acid Bacteria, Rapeseed Meal, Total Mixed Ration, Yeast

## Abstract

**Objective:**

This study aimed to determine the appropriate supplementation level of lactic acid bacteria (LAB; *Lactobacillus plantarum* and *Bacillus clausii*), yeast (*Saccharomyces cariocanus* and *Wickerhamomyces anomalus*) for degrading free gossypol and glucosinolate in the fermented total mixed ration (TMR) containing cottonseed meal (CSM) or rapeseed meal (RSM), to improve the utilization efficiency of these protein sources.

**Methods:**

For LAB, *L. plantarum* or *B. clausii* was inoculated at 1.0×10^8^, 1.0×10^9^, 1.0×10^10^, and 1.0×10^11^ colony-forming unit (CFU)/kg dry matter (DM), respectively. For yeast, *S. cariocanus* or *W. anomalus* was inoculated at 5×10^6^, 5×10^7^, 5×10^8^, and 5×10^9^ CFU/kg DM, respectively. The TMR had 50% moisture and was incubated at 30°C for 48 h. After fermentation, the chemical compositions, and the contents of free gossypol and glucosinolate were determined.

**Results:**

The results showed that the concentration of free gossypol content was reduced (p<0.05), while that of the crude protein content was increased (p<0.05) in the TMR containing CSM inoculated by *B. clausii* (1×10^9^ CFU/kg DM) or *S. cariocanus* (5×10^9^ CFU/kg DM). Similarly, the content of glucosinolate was lowered (p<0.05) and the crude protein content was increased (p<0.05) in TMR containing RSM inoculated with *B. clausii* (1×10^10^ CFU/kg DM) or *S. cariocanus* (5×10^9^ CFU/g DM).

**Conclusion:**

This study confirmed that inclusion of *B. clausii* with 1.0×10^9^ or 1.0×10^10^ CFU/kg DM, or *S. cariocanus* (5×10^9^ CFU/kg DM) to TMR containing CSM/RSM improved the nutritional value and decreased the contents of anti-nutritional factors.

## INTRODUCTION

With the rising demand for protein in the feed industry and the rising cost of soybean meal (SBM), it is becoming increasingly necessary to substitute other sources of protein. Therefore, the interest in research for alternative plant proteins to replace SBM in animal nutrition has grown. Two products that we are interested in are cottonseed meal (CSM) and rapeseed meal (RSM) because they are locally available and lower cost than SBM (on protein basis). Cottonseed meal is a by-product of cottonseed oil extraction, which contains approximately 34% to 40% of crude protein (CP), 11% of crude fiber (CF), as well as vitamin B and organic phosphorus [1]. Nevertheless, the use of CSM in animal diet is restricted due to the presence of free gossypol (FG), a toxic pigment which may have adverse effects on animals’ growth, reproduction, intestinal development, and lead to internal organ abnormalities [2,3]. Rapeseed meal is a by-product of rapeseed crushing after the oil extraction process and contains high protein level (34% to 38%) with a well-balanced amino acid composition and 25% to 30% neutral detergent fiber (NDF) [4]. Rapeseed meal protein is nutritionally comparable to soy protein and has more S-amino acids than many other plant proteins [5]. Nevertheless, the inclusion of RSM in animal diets is also limited due to anti-nutritional factors and high fiber levels [6]. Although RSM has a nutritional value comparable to SBM, it contains glucosinolate, sinapine, and the derivatives taninin and phytic acid and CF which may negatively affect animals' growth performance, health, and general welfare [7].

Several approaches have been used to decrease the anti-nutritional factors of CSM, such as calcium hydroxide [8], chemical treatment with ferrous sulfate [9], and microbial fermentation [10]. To reduce the anti-nutritional factors of RSM, methods such as inactivation of myrosinase, solvent extraction, steam removal, and liquidation have been applied. Still, such methods also have some disadvantages including loss of protein, high expense, commercial relative unimportance, and environmental pollution [11]. Fermented total mixed ration (FTMR) is an effective way to optimize nutrient utilization and feed storage life. Fermentation of total mixed ration (TMR) generated by microorganisms has been broadly accepted and commonly employed to enhance the feed quality [12]. An additive combination of TMR containing *Lactobacillus casei* (*L. casei*) TH14 with fermented sugarcane bagasse had significant effects on mid-lactation Holstein Friesian cows' intake, digestibility, rumen ecology, and milk output [13]. Fermenting feed with microbes is viewed as a promising solution [14,15], as it may be effective in reducing anti-nutritional components and increasing the amino acid content [16]. However, few studies tested if microbial fermentation can improve the nutritive value of the feed by enhancing the bioavailability of nutrients and decreasing the contents of anti-nutritional factors [17]. In China, fermented feed is usually manufactured by fermentation with an aim to reduce anti-nutritional factors in feed components such as CSM [18] and RSM [19].

Using lactic acid-producing bacteria (LAB) is an efficient method to reduce the contents of anti-nutritional elements in CSM and increase its nutritional value [20,21]. For example, Tang et al [22] reported that fermentation with *Bacillus subtilis* (*B. subtilis*) BJ-1 could reduce the amount of FG in CSM and that dietary inclusion of fermented CSM at a rate of 12% can promote the immunity and growth performance of animals. Previously published research has demonstrated that substituting CSM fermented by *B. subtilis* BJ-1 for SBM enhanced the growth performance and intestinal morphology while increasing the abundance of beneficial bacteria of broiler chickens [16,23]. According to Cherdthong et al [24], *L. casei* TH14 in combination with molasses or molasses plus cellulose produces superior outcomes by preventing CP degradation during fermentation, while increasing the digestibility of dry matter (DM) and organic matter, the rumen bacterial population, and concentration of propionic acid. *Lactobacillus plantarum* (LP) has been supplemented to TMR with silage and had improved rumen fermentation characteristics [25]. *Bacillus clausii* (*B. clausii*) is a gram-positive spore-forming microorganism, when administered in sufficient amounts, confers health advantages on the host [26].

Yeast is abundant in nature and easy to be cultured in large quantities [17]. *Saccharomycetes* has been widely used to reduce the contents of anti-nutritional factors of diet through fermentation and phytic acid degradation with phytase [27]. Additionally, yeast may increase the CP and mineral content of plant-based meals [28]. Most researchers have conducted their studies using *Saccharomyces cerevisiae* (S. cerevisiae). Although S. cerevisiae has numerous advantages, several drawbacks have been observed, especially low cell biomass [29]. Under aerobic circumstances, S. cerevisiae ferment alcohol rather than produces biomass [30]. This limits animals' access to nutritious yeast biomasses like protein, vital amino acids, and vitamins. As a result, it is critical to broaden the field of research and to strengthen the study of the use of additional yeast strains. Due to the limited amount of information available, *Saccharomyces cariocanus* (*S. cariocanus*) and *Wickerhamomyces anomalus* (*W. anomalus*) may be alternative options. However, little information is available on the fermented TMR containing CSM or RSM with LAB (e.g., *L. plantarum* and *B. clausii*) or yeast (e.g. *S. cariocanus* and *W. anomalus*) supplementation. The objectives of this research were to select the suitable level of LAB (*L. plantarum* and *B. clausii*) and yeast (*S. cariocanus* and *W. anomalus*), and to assess the effects of inoculants on the chemical compositions and anti-nutritional factors in fermented TMR. We hypothesized that the inoculation of LAB or yeast at an appropriate level to CSM or RSM might reduce the anti-nutritional factors while increasing the nutritional value.

## MATERIALS AND METHODS

### Experimental design and treatments

The experiment was performed from June to Dec 2020 at Laboratory of Ruminant Feed Nutrition Innovation, Institute of Feed Research of Chinese Academy of Agricultural Sciences, Beijing, China. One-way design was used to evaluate the effect of four different inoculum dosage levels of LAB or yeast on anti-nutritional factors and chemical composition of FTMR containing CSM or rapeseed. Treatments including control with no inoculant; F control, fermented control without inoculum; LP1, inoculated with *L. plantarum* with 1×10^8^ colony-forming units (CFU)/kg DM; LP2, inoculated with *L. plantarum* with 1×10^9^ CFU/kg DM; LP3, inoculated with *L. plantarum* with 1×10^10^ CFU/kg DM; LP4, inoculated with *L. plantarum* with 1×10^11^ CFU/kg DM, or BC1, inoculated with *B. clausii* with 1×10^8^ CFU/kg DM; BC2, inoculated with *B. clausii* with 1×10^9^ CFU/kg DM; BC3, inoculated with *B. clausii* with 1×10^10^ CFU/kg DM; BC4, inoculated with *B. clausii* with 1×10^11^ CFU/kg DM. Similarly treatments of inoculum dosage levels of yeast including control with no inoculant; F control, fermented control without inoculum; SC1, inoculated with *S. cariocanus* with 5×10^6^ CFU/kg DM; SC2, inoculated with *S. cariocanus* with 5×10^7^ CFU/kg DM; SC3, inoculated with *S. cariocanus* with 5×10^8^ CFU/kg DM; SC4, inoculated with *S. cariocanus* with 5×10^9^ CFU/kg DM; or WA1, inoculated with *W. anomalus* with 5×10^6^ CFU/kg DM; WA2, inoculated with *W. anomalus* with 5×10^7^ CFU/kg DM; WA3, inoculated with *W. anomalus* with 5×10^8^ CFU/kg DM; WA4, inoculated with *W. anomalus* with 5×10^9^ CFU/kg DM.

### Experimental materials

*L. plantarum*, *B. clausii*, *S. cariocanus*, and *W. anomalus* used in this study were purchased from a local company (Gaotang Huanong Bioengineering Co. Ltd., Shandong, China). The CSM and RSM used as fermentation substrate were collected from a local feed manufacturer (Dadi Feed Company, Chengdu, Sichuang, China).

### Real-time quantitative polymerase chain reaction analysis of inoculants

Total bacterial and yeast primers were used to quantify the LAB and yeast, respectively. The primer for total bacterial detection was designed based on 16S rRNA V4 region (515F: GTGYCAGCMGCCGCGGTAA and 806R: GGACTACN VGGGTWTCTAAT). The primer for total yeast was designed based on ITS region (ITS1F CTTGTCATTTAGGA AGTAA and ITS2R GCTGCGTTTCATCGATGATGC). In the same quantitative polymerase chain reaction (qPCR) system, the amount of both sample and standard sample was 1 μL. Then, the samples were mixed, centrifuged, and divided into 96 well PCR plates. Each sample had three replications for each gene. An initial denaturation at 95°C for 10 minutes was followed by 40 denaturation cycles at 95°C for 20 seconds, followed by annealing at 60°C for 30 seconds. Extractions of DNA and RNA were conducted using Power Soil DNA extraction kit: 142579, Qiagen and Mini RNA extraction kit 217004, Qiagen following the manufacturer's instructions of protocol (Life Technologies, Beijing, China). The concentrations of DNA and RNA of LAB were determined together, and the units are shown as copies/g.

### Preparation and fermentation of TMR containing CSM and RSM with inoculants

**Figure 1:**
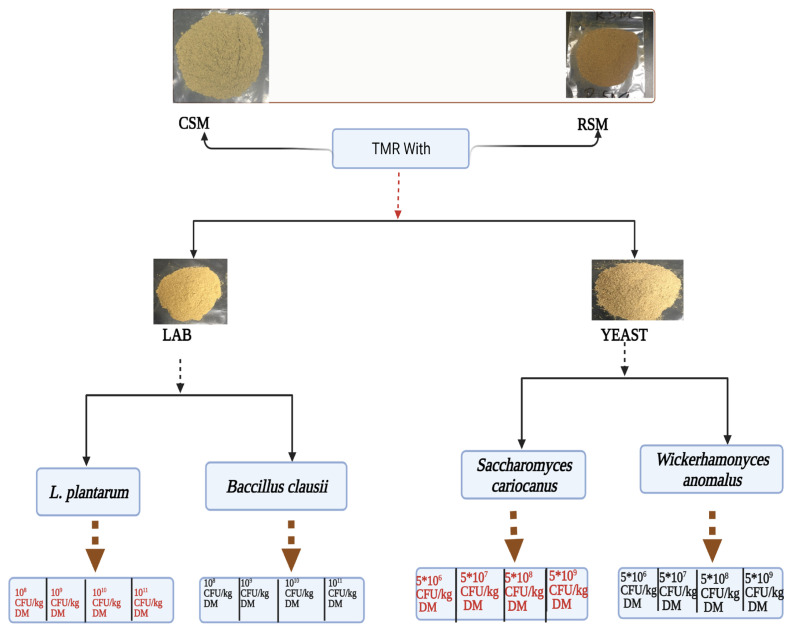
Schematic illustration of the design of LAB and yeast of inoculating TMR with CSM or RSM. TMR, total mixed ration; CSM, cottonseed meal; RSM, rapeseed meal; LAB, lactic acid bacteria; CFU, colony-forming units; DM, dry matter.

The CSM and RSM were used as a fermentation substrate in TMR. The *L. plantarum* or *B. clausii* were added into TMR containing CSM or RSM at 1.0×10^8^, 1.0×10^9^, 1.0×10^10^, and 1.0×10^11^ CFU/kg DM, respectively as shown in Figure 1. *S. cariocanus* or *W. anomalus* were added into TMR containing CSM or RSM at 5×10^6^, 5×10^7^, 5×10^8^, and 5×10^9^ CFU/kg DM, respectively [31,32]. The TMR was mixed thoroughly and uniformly, and moisture content was adjusted to 50%. The ingredients and chemical compositions of the diets are shown in Table 1. A vacuum sealer machine was used to remove air from the fermentation plastic bags. TMR were incubated in an incubator at 30°C for 48 h. Following completion of fermentation, the inoculated samples were dried at 65°C for 48 h, cooled, and ground to a size of 2 mm. The dried samples were transferred into new plastic bags and stored at −20°C for later analysis. Triplicate plastic bags were used for each treatment.

### Measurements

### Chemical composition

TMR with fermented CSM or RSM, fermented control (control group that was not inoculated but fermented) and original control (neither inoculated nor fermented) were made and prepared for subsequent analysis. Samples were ground to pass through a 1-mm sieve size for analysis of DM, CP, and ether extract (EE) according to AOAC [33]. According to Van Soest et al [34], NDF and acid detergent fiber (ADF) were determined.

### Anti-nutritional factors analysis

The FG was determined using the official method of the American Oil Chemists Society [35]. Free gossypol was determined by the presence of 3-amino-1-propanol, a mixture of isopropyl alcohol and n-hexane was used to extract FG, and aniline was used to convert gossypol to aniline cotton phenol, and the colorimetric determination was carried out at the maximum absorption of the spectrophotometer at the wavelength of 440 nm of a spectrophotometer. Two grams of TMR with CSM sample was put in a 250 mL Erlenmeyer flask with stopper, 20 glass beads, and pipette. The tube was filled with 50 mL of solvent, closed the bottle, put it in the shaker, and was oscillated for 1 h. A dry filter was used and then was covered with funnel glass to reduce the solvent volatilization. The first few drops of filtrate were discarded and the remaining was collected using 100 mL Erlenmeyer flask with a stopper.

Calculation formula


$$X=\frac{A\times 1,250\times 1,000}{a\times m\times V}=\frac{A\times 1.25}{amV}\times 10^{6}$$

In the formula: X = FG content (mg/kg); A = correct the absorbance; m = sample quality (g); V = the volume of filtrate for determination (mL); a = mass absorption coefficient, FG is 62.5 cm^−1^g^−1^L.

Correspondingly, glucosinolates of the TMR with RSM were determined using palladium chloride [36]. Briefly, 0.2 g TMR with RSM was powdered in a mortar and added to a graduated test tube containing 10 mL boiling water. The mixtures were thoroughly shaken and heated for 30 minutes in water bath before being diluted to ten milliliters. Following centrifugation, 2 mL of TMR with RSM extract suspension was pipetted to a graduated tube containing 4 mL of 0.15% of sodium carboxymethyl cellulose and shaken well. Then, 2 mL of 8 mmol/L palladium chloride color was added. After vigorous stirring, the mixed solutions were kept at 22°C±3°C for 2 h. The absorption at 540 nm (A) was determined using sodium carboxymethyl cellulose as the reference material and a blank solution as the standard solution. The glucosinolate content was determined using absorbance A, which is proportional to the glucosinolate content as ascertained by the standard curve. Standard curve: A = Kx+b.


Glucosinolate content X (μmol/g)=(A-b)/k.

### Statistical analysis

Data were analyzed with SPSS version 23.0 (IBM Corp., Armonk, NY, USA). One-way analysis of variance was used to analyze the effects of inoculants on the chemical composition and anti-nutritional factors. The significance of differences between mean values was assessed using Tukey's multiple comparisons. Differences between the treatments were considered significant if p<0.05, and results were visualized using GraphPad Prism version 8.3 (San Diego, CA, USA).

## RESULTS

### Real-time quantitative polymerase chain reaction analysis

**Figure 2:**
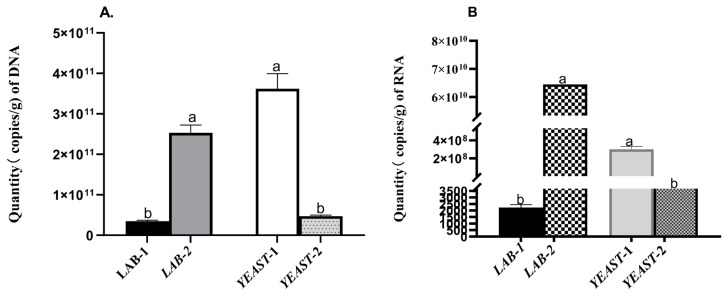
Comparison of the quantities (copy/g) of (A) DNA and (B) RNA of LAB and yeast. The DNA and RNA levels were determined by real-time PCR. LAB-1, *Lactobacillus plantarum*; LAB-2, *Bacillus clausii*; Yeast-1, *Saccharomyces cariocanus*; Yeast-2, *Wickerhamomyces anomalus*; PCR, polymerase chain reaction.

The DNA and RNA levels of four strains were determined by real-time PCR. *B. clausii* had higher (p<0.05) DNA and RNA levels compared to *L. plantarum*. In addition, *S. cariocanus* showed higher (p<0.05) DNA and RNA levels than *W. anomalus* (Figure 2A and 2B).

### Chemical composition of fermented TMR with CSM or RSM

In the fermented TMR containing CSM with LAB supplementation, the contents of DM, EE, and NDF did not differ (p>0.05) among treatments (Table 2). Fermentation with *L. plantarum* and *B. clausii* increased CP content (p<0.05). All groups inoculated with *B. clausii* at 1×10^9^ CFU/kg DM showed the highest CP content (15.24%). The CP contents of BC1 (*B. clausii* with 1×10^8^ CFU/k g DM) and BC2 (*B. clausii* with 1×10^9^ CFU/kg DM) were higher (p<0.05) than that of control group. The ADF of LP4 (*L. plantarum* with 1×10^11^ CFU/kg DM) and BC4 (*B. clausii* with 1×10^11^ CFU/k g DM) showed the lowest reduction (p<0.05) than that of control group. In the fermented TMR containing CSM with yeast supplementation, the contents of DM, CP, EE, NDF, and ADF did not differ (p>0.05) among treatments (Table 3). Nonetheless, the CP content of SC4 (*S. cariocanus* with 5×10^9^ CFU/kg DM) was numerically higher than that of other treatments.

As shown in Table 4, in the fermented TMR containing RSM with LAB supplementation, the DM, CP, and NDF contents differ (p<0.05) among treatments. The treatment inoculated with *B. clausii* at 1×10^10^ CFU/kg DM showed the highest CP content (p<0.05). In the meantime, the treatments inoculated with *B. clausii* at 1×10^9^ and 1×10^11^ CFU/kg DM showed the highest NDF content (p<0.05). In the fermented TMR containing RSM with yeast supplementation, only the CP and NDF contents differ (p<0.01) among treatments, which were lowest in the control group (Table 5).

### Anti-nutritional factors of TMR with CSM or RSM

As shown in Table 6, the detoxification efficiency of FG varied with different types and levels of strains. Microbial inoculation decreased (p<0.05) FG levels during the fermentation. The detoxification efficiency of *B. clausii* were much higher than that of *L. plantarum*. Compared with control group, the levels of glucosinolate were lower (p<0.05) in BC4 (*B. clausii* with 1.0×10^11^ CFU/kg DM), BC3 (*B. clausii* with 1.0×10^10^ CFU/kg DM), BC2 (*B. clausii* with 1.0×10^9^ CFU/kg DM), BC1 (*B. clausii* with 1.0×10^8^ CFU/kg DM), LP1 (*L. plantarum* with 1.0×10^8^ CFU/kg DM), LP2 (*L. plantarum* with 1.0×10^9^ CFU/kg DM), LP3 (*L. plantarum* with 1.0×10^10^ CFU/kg DM), and LP4 (*L. plantarum* with 1.0×10^11^ CFU/kg DM), but not compared to the fermented control. *B. clausii* inoculation with 1.0×10^11^ CFU/kg DM showed the lowest glucosinolates content (18.45 to 3.86) compared with all treatments inoculated with *L. plantarum* or *B. clausii*. In comparison, the RSM-based TMR with *B. clausii* at 1.0×10^10^ CFU/kg DM decreased glucosinolate content (18.45 to 5.86).

On the other hand, there were significant differences (p< 0.05) in the content of FG with *S. cariocanus* and *W. anomalus* (Table 7). The inoculation with *S. cariocanus* or *W. anomalus* led to significant reduction of FG content (p<0.01). Relative to the other, SC3 (*S. cariocanus* with 5×10^8^ CFU/kg DM), SC4 (*S. cariocanus* with 5×10^9^ CFU/kg DM), WA2 (*W. anomalus* with 5×10^7^ CFU/kg DM), and WA4 (*W. anomalus* with 5×10^9^ CFU/kg DM), showed improved degradation levels. In Table 7, the treatments were varied with varying levels of degradation of glucosinolate. *S. cariocanus* or *W. anomalus* significantly decreased the glucosinolate level (p<0.05) compared to the control group. However, in comparison to the control group, SC4 (*S. cariocanus* with 5×10^9^ CFU/kg DM) and WA4 (*W. anomalus* with 5×10^9^ CFU/kg DM) showed better reductions in glucosinolate levels than the control group, except for the fermented control group. The biological meaning of our result is that we have not seen any nature and size of relevant biological changes or differences between the results. Therefore, that means our result has not shown any biological effect of chemical composition on TMR

## DISCUSSION

The quantity of both RNA and DNA provides an indication of active cells, starved or dead cells. Both DNA/RNA of *L. plantarum* were lower compared with *B. clausii* examined by real-time PCR, indicating that most *L. plantarum* was no longer active. Our results concur the finding that *L. plantarum* incubated in glycerol 2-phosphate buffer possessed extremely low RNA/DNA ratios [37]. Similarly, the number of copies of both DNA & RNA of *S. cariocanus* also was higher than that of *W. anomalus*.

In the current study, the supplementation of different levels of LAB or yeast effectively decreased the anti-nutritional factors and increased nutritional value of fermented TMR containing CSM or RSM. The supplementation of *L. plantarum* or *B. clausii* to TMR with CSM increased the CP content, consistent with a previous study indicating solid-state fermentation (SSF) using *B. subtilis* BJ-1 increased CP content from 46.5% to 50.5% [22]. Similarly, when CSM was inoculated with *B. subtilis* ST-141 and *Saccharomycetes* N5, CP increased from 49.8% to 51% [38]. On the other hand, inoculation with *L. plantarum* or *B. clausii* to TMR with RSM also increased the CP content. The highest CP value (13.82%) was shown in BC3 (*B. clausii* with 1×10^10^ CFU/kg DM). These results agree with Fazhi et al [39] who found that *L. plantarum* and *B. subtilis* increased the CP content of fermented RSM. Similar results were also observed when *L. fermentum* and *B. subtilis* have been inoculated [40]. The increase in CP content could be due to the multiplication of the microorganisms responsible for the rise in protein used by the high availability of soluble carbohydrates.

Although there was no significant difference among the DM contents of both fermented TMR with CSM/RSM, it slightly reduced when fermented TMRs with CSM or RSM were inoculated with *L. plantarum*, *B. clausii*, *S. cariocanus* and *W. anomalus* at different levels. These results may be due to the loss of DM in fermented TMR with CSM or RSM, which causes a relative rise in the concentration of these nutrients. A rise in CP content may be a result of decrease in DM content. Our findings are consistent with the study of Wang et al [38] who discovered that when CSM was inoculated with *B. subtilis* ST-141 and *Saccharomycetes* N5, the DM content was decreased. In agreement with the present study, SSF led to reduced DM content of RSM [41]. The decrease in DM content could be a result of a decreased number of microorganisms utilizing carbohydrate consumption as an energy source for growth and survival. According to Rozan et al [42], reducing DM content during fermentation may account for the increase in CP, the content of which was increased following fermentation. Besides, Schmidt et al [43] reported that an addition of homolactic *L. plantarum*, *Enterococcus faecium*, and heterolactic *L. Brevis* in ensilage of sugarcane, which indicated a domination of homolactic fermentation, with an rise in lactic acid and ethanol content, and reduced DM (43 g/kg DM; 186 g/kg DM, and 272 g/kg DM, respectively) relative to control (36 g/kg DM; 144 g/kg DM, and 144 g/kg DM respectively). The increase in NDF showed that the inoculated microbial dosage levels of the treated TMR with RSM were insufficient to control these rises in NDF of TMR with RSM. Our result were consistent with previous studies [44] that reported CF content was often elevated or slightly reduced after fermentation. Moreover, Pedroso et al [45] reported that NDF and ADF levels were increased during silage processing in the DM loss of soluble carbohydrates such as gasses and effluents. The increase in NDF may be attributed to the loss of DM, degradation of glucosinolates, and inadequate fiber hydrolysis during the fermentation process of TMR with RSM. The possible accumulation of acid, alkaline and neutral detergent insoluble substances during SSF can also be stated as another cause for this observation [46,47], thus overstating the NDF and ADF levels. The increases in NDF may suggest that the inoculum dosage of *L. plantarum*, *B. clausii*, *S. cariocanus*, *W. anomalus* were insufficient to control these increases of NDF and DM loss of TMR with RSM.

Our result of FG degradation was lower than that reported by Tang et al [22], which reduced FG in solid-state fermented cotton meal from 0.82 to 0.21 g/kg. Similarly, FG reduced from 90 to 30 mg/kg in the study by Xiong et al [48]. Sun et al [49] found fermented CSM by *B. subtilis* BJ-supplement significantly reduced FG level and increased CP level. Comparably, Wang et al [38] stated that the fermented CSM by *B. subtilis* ST-141 and *Saccharomycetes* N5 dramatically reduced the FG level (from 820 to 346 mg/kg). But the result in our study is higher than that reported by Duodu et al [50] where FG level was reduced by approximately 17% during short-term fermentation with yeast (*S. cerevisiae*). However, a reduction in FG level was shown in TMR with CSM during microbial fermentation. In addition to the nutritional improvement of TMR with CSM, the level of FG in TMR were significantly lowered due to fermentation with varying dosage levels of microbial strains. The reduction of FG may be the result of gossypol being bound to microbial enzymes that work to break down gossypol during the fermentation of TMR with CSM. For these reasons, considering the decreased FG level and increased CP content, SC4 (*S. cariocanus* with 5×10^9^ CFU/kg DM) and BC2 (*B. clausii* with 1.0×10^9^ CFU/kg DM) were selected as the most suitable strains for the subsequent experiment.

Our result of glucosinolate degradation is consistent with previous study, which decreased glucosinolate content from 41.91 to 23.86 μmol/g [51]. In line with the earlier findings of Ahmed et al [52], the current findings revealed that the increased protein content by solid-state fermentation with *Lactobacillus salivarius* was from 41.2% to 42.2%, and the reduction in glucosinolates was from 22 to 13.6 mmol/g. Likewise, it was reported that fermentation of RSM with *Lactobacillus fermentum*, *B. subtilis*, *S. cerevisiae*, and *Enterococcus faecium* decreased the isothiocyanates (derivatives of glucosinolates) and increased the CP content [40,41]. Reduced glucosinolates and increased CP content were observed during fermentation of RSM by [11,53]. The loss of glucosinolates led to the creation of glucose and sulphur molecules through microbial enzymes during fermentation [6]. Considering the glucosinolate-degrading ability, increased protein, and decreased NDF, both BC3 (*B. clausii* with 1.0×10^10^ CFU/kg DM) and SC4 (*S. cariocanus* with 5×10^9^ CFU/kg DM) were selected to conduct the next experiment.

## CONCLUSION

This study demonstrates that *B. clausii* with 1.0×10^9^/1.0×10^10^ CFU/kg DM and *S. cariocanus* with 5×10^9^ CFU/kg DM capable of degrading anti-nutritional factors as well as improving the nutritional value of fermented TMR with CSM/RSM is beneficial. We found the decrease in the concentrations of anti-nutritional factors and enhancement of nutritional value of a fermented TMR containing CSM/RSM, and thus recommended that this fermented source of protein can be used as an appropriate alternative to SBM in ruminant diets. Further studies need to be conducted on the feed nutrient values and the safety of the original fermented groups, the mixed of strains of LAB and yeast, different dosage levels, and rate of application for optimizing the beneficial effects for the development of the nutritional value and anti-nutritional factors of the next generation of the TMR with CSM/RSM inoculant.

## Figures and Tables

**Table 1:** Ingredients and nutrient composition of TMR with CSM or RSM (% of DM)

Items	TMR with CSM	TMR with RSM
Ingredient		
Corn	33.55	33.48
Wheat bran	12	12
CSM	10	0
RSM	0	10
Whole corn silage	20	20
Cornstalk	20	20
Fat powder	0.3	0.3
Urea	0.15	0.22
Premix	4	4
Total	100	100
Chemical composition		
DM (fresh basis)	51.77	52.72
CP	13.61	12.68
EE	2.37	1.910
NDF	38.17	34.32
ADF	20.69	17.26

TMR, total mixed ration; CSM, cottonseed meal; RSM, Rapeseed meal; DM, dry matter; CP, crude protein; EE, ether extract; NDF, neutral detergent fiber; ADF, acid detergent fiber.

**Table 2:** Effect of inoculated *Lactobacillus plantarum* or *Bacillus clausii* on chemical composition of TMR with CSM (% DM)

Items	Control	F control	*Lactobacillus Plantarum* ^1)^				*Bacillus clausii* ^1)^				SEM	p-value
	
			LP1	LP2	LP3	LP4	BC1	BC2	BC3	BC4		
DM (fresh basis)	51.77	47.53	50.09	51.06	51.65	50.69	47.17	46.53	48.51	50.19	0.499	0.107
CP	13.61^a^	14.64^ab^	14.09^ab^	14.29^ab^	14.32^ab^	14.23^ab^	15.05^b^	15.24^b^	14.61^ab^	14.63^ab^	0.116	0.009
EE	2.37	2.57	2.54	2.31	3.24	2.70	3.33	3.04	2.69	3.17	0.141	0.777
NDF	38.17	35.34	35.14	33.34	34.47	34.92	35.74	37.48	35.57	37.32	0.401	0.162
ADF	20.69^a^	17.77^ab^	16.67^b^	16.34^b^	15.38^b^	15.99^b^	16.67^b^	17.72^ab^	16.48^b^	16.39^b^	0.329	0.007

TMR, total mixed ration; CSM, cottonseed meal; DM, dry matter; SEM, pooled standard error of means; CP, crude protein; EE, ether extract; NDF, neutral detergent fiber; ADF, acid detergent fiber.

1) Treatments including control with no inoculant; F control, fermented control without inoculum; LP1, *Lactobacillus plantarum* with 1×10^8^ CFU/kg DM; LP2, *Lactobacillus plantarum* with 1×10^9^ CFU/kg DM; LP3, *Lactobacillus plantarum* with 1×10^10^ CFU/kg DM; LP4, *Lactobacillus plantarum* with 1×10^11^ CFU/kg DM; BC1, *Bacillus clausii* with 1×10^8^ CFU/kg DM; BC2, *Bacillus clausii* with 1×10^9^ CFU/kg DM; BC3, *Bacillus clausii* with 1×10^10^ CFU/kg DM; BC4, *Bacillus clausii* with 1×10^11^CFU/kg DM; CFU, colony-forming units.

a,b Means in the same row with different superscripts differed (p<0.05).

**Table 3:** Effect of yeast inoculation levels on chemical composition of TMR with CSM (% DM basis)

Items	Control	F control	*Saccharomyces cariocanus* ^1)^				*Wickerhamomyces anomalus* ^1)^				SEM	p-value
	
			SC1	SC2	SC3	SC4	WA1	WA2	WA3	WA4		
DM (fresh basis)	51.77	47.53	49.17	49.28	48.44	48.09	53.09	47.37	47.99	49.36	0.495	0.146
CP	13.61	14.64	14.35	14.73	15.14	15.96	15.11	15.27	15.54	14.55	0.181	0.180
EE	2.37	2.57	3.45	2.62	2.25	2.59	2.45	2.30	2.73	2.42	0.132	0.793
NDF	38.17	35.34	34.02	32.95	34.21	34.30	35.35	34.43	34.62	33.02	0.405	0.192
ADF	20.69	17.77	16.91	19.06	17.44	18.76	17.81	17.10	18.50	17.11	0.352	0.374

TMR, total mixed ration; CSM, cottonseed meal; DM, dry matter; SEM, pooled standard error of means; CP, crude protein; EE, ether extract; NDF, neutral detergent fiber; ADF, acid detergent fiber.

1) Treatments including control with no inoculant, F control, fermented without inoculum; SC1, *Saccharomyces cariocanus* with 5×10^6^ CFU/kg DM; SC2, *Saccharomyces cariocanus* with 5×10^7^ CFU/kg DM; SC3, *Saccharomyces cariocanus* with 5×10^8^ CFU/kg DM SC4; *Saccharomyces cariocanus* with 5×10^9^ CFU/kg DM; WA1, *Wickerhamomyces anomalus* with 5×10^6^ CFU/kg DM; WA2, *Wickerhamomyces anomalus* with 5×10^7^ CFU/kg DM; WA3, *Wickerhamomyces anomalus* with 5×10^8^ CFU/kg DM; WA4, *Wickerhamomyces anomalus* with 5×10^9^ CFU/kg DM; CFU, colony-forming units.

**Table 4:** Effect of Inoculated *Lactobacillus Plantarum* or *Bacillus clausii* on chemical composition of TMR with RSM (% DM basis)

Items	Control	F control	Lactobacillus plantarum^1)^				Bacillus clausii^1)^				SEM	p-value
	
			LP1	LP2	LP3	LP4	BC1	BC2	BC3	BC4		
DM (fresh basis)	52.72	49.90	49.72	48.84	49.61	50.61	49.31	49.32	48.34	49.70	0.344	0.289
CP	12.68^b^	13.33^ab^	13.49^ab^	13.62^ab^	13.34^ab^	13.23^ab^	13.35^ab^	13.31^ab^	13.82^a^	13.43^ab^	0.071	0.045
EE	1.91	2.39	1.95	1.97	2.00	1.54	2.31	2.09	2.08	1.975	0.100	0.902
NDF	34.32^b^	38.61^ab^	37.26^ab^	37.91^ab^	37.83^ab^	37.00^ab^	38.29^ab^	39.22^a^	37.78^ab^	39.25^a^	0.342	0.043
ADF	17.26	19.64	18.95	22.13	19.91	18.95	20.48	19.62	19.14	19.88	0.327	0.127

TMR, total mixed ration; RSM, rapeseed meal; DM, dry matter; SEM, pooled standard error of means; CP, crude protein; EE, ether extract; NDF, neutral detergent fiber; ADF, acid detergent fiber.

1) Treatments including control with no inoculant, F control, fermented without inoculum; LP1, *Lactobacillus plantarum* with 1×10^8^ CFU/kg DM; LP2, *Lactobacillus plantarum* with 1×10^9^ CFU/kg DM; LP3, *Lactobacillus plantarum* with 1×10^10^ CFU/kg DM; LP4, *Lactobacillus plantarum* with 1×10^11^ CFU/kg DM; BC1, *Bacillus clausii* with 1×10^8^ CFU/kg DM; BC2, *Bacillus clausii* with 1×10^9^ CFU/kg DM; BC3, *Bacillus clausii* with 1×10^10^ CFU/kg DM; BC4, *Bacillus clausii* with 1×10^11^ CFU/kg DM; CFU, colony-forming units.

a,b Means in the same row with different superscripts differed (p<0.05).

**Table 5:** Effect of yeast inoculation levels on chemical composition of fermented TMR with RSM (% DM basis)

Items	Control	F control	*Saccharomyces cariocanus* ^1)^				*Wickerhamomyces anomalus* ^1)^				SEM	p-value
	
			SC1	SC2	SC3	SC4	WA1	WA2	WA3	WA4		
DM (fresh basis)	52.72	49.90	49.09	48.79	48.76	48.72	48.99	49.71	48.44	48.13	0.351	0.160
CP	12.68^b^	13.33^ab^	13.46^ab^	13.55^ab^	13.66^a^	13.94^a^	13.59^a^	13.62^a^	13.69^a^	14.11^a^	0.082	0.002
EE	1.91	2.39	1.77	2.08	2.16	1.94	2.31	1.50	2.01	2.52	0.089	0.306
NDF	34.32^b^	38.61^ab^	36.57^ab^	39.66^a^	37.97^ab^	37.38^ab^	38.44^ab^	38.89^ab^	37.77^ab^	39.82^a^	0.385	0.033
ADF	17.26	19.64	20.39	19.96	20.90	18.99	19.90	19.85	20.00	19.79	0.243	0.063

TMR, total mixed ration; RSM, rapeseed meal; DM, dry matter; SEM, pooled standard error of means; CP, crude protein; EE, ether extract; NDF, neutral detergent fiber; ADF, acid detergent fiber.

1) Treatments including control with no inoculant, F control, fermented control without inoculum; SC1, *Saccharomyces cariocanus* with 5×10^6^ CFU/kg DM; SC2, *Saccharomyces cariocanus* with 5×10^7^ CFU/kg DM; SC3, *Saccharomyces cariocanus* with 5×10^8^ CFU/kg DM; SC4, *Saccharomyces cariocanus* with 5×10^9^ CFU/kg DM; WA1, *Wickerhamomyces anomalus* with 5×10^6^ CFU/kg DM; WA2, *Wickerhamomyces anomalus* with 5×10^7^ CFU/kg DM; WA3, *Wickerhamomyces anomalus* with 5×10^8^ CFU/kg DM; WA4, *Wickerhamomyces anomalus* with 5×10^9^ CFU/kg DM; CFU, colony-forming units.

a,b Means in the same row with different superscripts differed (p<0.05).

**Table 6:** Effect of lactic acid bacteria strains on free gossypol/glucosinolate degradation (as-DM basis)

Items	Control	F control	*Lactobacillus plantarum* ^1)^				*Bacillus clasusii* ^1)^				SEM	p-value
	
			LP1	LP2	LP3	LP4	BC1	BC2	BC3	BC4		
CSM-based TMR												
FG (μmol/g)	92.94^a^	77.18^b^	73.71^b^	75.84^b^	75.13^b^	68 87^bc^	57.36^c^	56.34^c^	54.05^c^	53.49^c^	2.60	<0.001
RSM-based TMR												
GSLs (μmol/g)	18.45^a^	7.71^b^	8.13^b^	7.11^b^	5.91^bc^	6.20^bc^	6.86^b^	6.21^bc^	5.86^bc^	3.86^c^	0.74	<0.001

DM, dry matter; SEM, pooled standard error of means; CSM, cottonseed meal; TMR, total mixed ration; FG, free gossypol; RSM, rapeseed meal; GSLs, glucosinolates.

1) Treatments including control with no inoculant, F control, fermented control without inoculum; LP1, *Lactobacillus plantarum* with 1×10^8^ CFU/kg DM; LP2, *Lactobacillus plantarum* with 1×10^9^ CFU/kg DM; LP3, *Lactobacillus plantarum* with 1×10^10^ CFU/kg DM; LP4, *Lactobacillus plantarum* with 1×10^11^ CFU/kg DM; BC1, *Bacillus clausii* with 1×10^8^ CFU/kg DM; BC2, *Bacillus clausii* with 1×10^9^ CFU/kg DM; BC3, *Bacillus clausii* with 1×10^10^ CFU/kg DM; BC4, *Bacillus clausii* with 1×10^11^ CFU/kg DM; CFU, colony-forming units.

a–c Means in the same row with different superscripts differed (p<0.05).

**Table 7:** Effect of yeast strains on free gossypol/glucosinolate degradation (as-DM basis)

Items	Control	F Control	*Saccharomyces cariocanus* ^1)^				*Wickerhamomyces anomalus* ^1)^				SEM	p-value
	
			SC1	SC2	SC3	SC4	WA1	WA2	WA3	WA4		
CSM based TMR												
FG (μmol/g)	92.94^c^	77.18^bc^	57.82^ab^	57.75^ab^	54.17^a^	52.23^a^	56.59^a^	50.64^a^	61.21^ab^	56.63^a^	2.55	<0.001
RSM-based TMR												
GSLs (μmol/g)	18.45^a^	7.71^b^	6.38^b^	6.87^b^	6.05^b^	5.97^b^	6.09^b^	8.57^b^	7.09^b^	6.02^b^	0.71	<0.001

DM, dry matter; SEM, pooled standard error of means; CSM, cottonseed meal; TMR, total mixed ration; FG, free gossypol; RSM, rapeseed meal; GSLs, glucosinolates.

1) Treatments including control with no inoculant, F control, fermented control without inoculum; SC1, *Saccharomyces cariocanu*s with 5×10^6^ CFU/kg DM; SC2, *Saccharomyces cariocanus* with 5×10^7^ CFU/kg DM; SC3, *Saccharomyces cariocanus* with 5×10^8^ CFU/kg DM; SC4, *Saccharomyces cariocanus* with 5×10^9^ CFU/kg DM; WA1, *Wickerhamomyces anomalus* with 5×10^6^ CFU/kg DM; WA2, *Wickerhamomyces anomalus* with 5×10^7^ CFU/kg DM; WA3, *Wickerhamomyces anomalus* with 5×10^8^ CFU/kg DM; WA4, *Wickerhamomyces anomalus* with 5×10^9^ CFU/kg DM; CFU, colony-forming units.

a–c Means in the same columns with different superscripts differed (p<0.05).
